# Maternal–Fetal Compatibility in Recurrent Pregnancy Loss

**DOI:** 10.3390/jcm13082379

**Published:** 2024-04-19

**Authors:** Isabel Cuadrado-Torroglosa, Juan A. García-Velasco, Diana Alecsandru

**Affiliations:** 1 IVIRMA Global Research Alliance, IVI Foundation, Instituto de Investigación Sanitaria La Fe (IIS La Fe), Avenida Fernando Abril Martorell, 106, Torre A, Planta 1^a^, 46026 Valencia, Spain; isabel.cuadrado@ivirma.com (I.C.-T.); juan.garcia.velasco@ivirma.com (J.A.G.-V.); 2 IVIRMA Global Research Alliance, IVIRMA Madrid, Av. del Talgo, 68, 28023 Madrid, Spain; 3 Department of Obstetrics and Gynaecology, Rey Juan Carlos University, Av. de Atenas, s/n, 28922 Alcorcón, Spain

**Keywords:** RPL, immunology, maternal–fetal interface, uNK cells, KIR, HLA

## Abstract

Nowadays, recurrent pregnancy loss (RPL) is an undesirable condition suffered by many patients of reproductive age. In this scenario, certain immune cell populations and molecules, involved in maternal–fetal compatibility, have emerged as factors related with the pathogenesis of RPL. Among them, uterine Natural Killer cells (uNKs) appear to be of great relevance. These cells are involved in numerous processes during pregnancy, such as the remodeling of uterine spiral arteries or the control of trophoblast invasion. These functions are regulated by the interactions that these cells establish with the extravillous trophoblast, mainly through their Killer Immunoglobulin-like Receptors (KIRs) and the Human Leukocyte Antigen-C (HLA-C) molecules expressed by the embryo. A high level of polymorphism has been reported for both molecules involved in this interaction, with some of the possible KIR–HLA-C combinations being associated with an increased risk of RPL. However, the complexity of the maternal–fetal interface goes beyond this, as other HLA molecules also appear to be related to this reproductive pathology. In this review, we will discuss the role of uNKs in pregnancy, as well as the polymorphisms and clinical implications of KIR–HLA-C binding. We will also address the involvement of other, different HLA molecules in RPL, and the current advice on the appropriate management of patients with ‘immunological mismatch’, thus covering the main aspects regarding the involvement of maternal–fetal compatibility in RPL.

## 1. Introduction

Recurrent pregnancy loss (RPL), also known as recurrent miscarriage (RM), is an undesirable reproductive outcome, faced by approximately 2.5% of women trying to conceive. This condition is defined as the occurrence of two or more spontaneous miscarriages, before weeks 20–24 of gestation, in clinically recognized pregnancies [1,2].

Currently, it is known that the primary cause of most miscarriages is embryonic aneuploidy, a condition closely linked to maternal age. In fact, chromosomal abnormalities are detected in around 40% of gestational losses in patients experiencing recurrent miscarriages [3]. Today, the application of certain techniques, such as preimplantation genetic testing (PGT), has reduced the probability of miscarriage in patients undergoing assisted reproduction treatments (ART), significantly increasing birth rates and improving the prognosis of couples facing previous gestational losses [1,4].

However, despite the numerous advances in the field, several pregnancies end in miscarriage, with no explanation that can be provided to patients as to the pathogenesis of certain cases of RPL. In fact, it is estimated that 50% of couples diagnosed with RPL are of unknow etiology. This is the main reason that prompts physicians and basic scientists alike to delve deeper into the possible causes of RPL, and to develop and tailor different treatments to suit each couple [1,5].

Beyond genetics, several factors have been pointed out as responsible of RPL: uterine abnormalities, endocrinological disorders, antiphospholipid syndrome, male factor, or even life habits [6]. However, recent studies in the field of reproductive immunology have highlighted another, sometimes underestimated, aspect that is widely implicated in the early stages of pregnancy and, consequently, in the entire development of a healthy gestation: the maternal–fetal interface and its immunological compatibility [7,8].

The maternal–fetal interface refers to the complex immunological microenvironment that is established between the mother and the embryo during pregnancy. It is in this milieu that the embryonic trophoblast cells come into contact with the maternal decidual and stromal cells, thus allowing the co-existence of two genetically different entities [9,10]. At this unique interface, key processes for pregnancy occur, such as, among others, the exchange of oxygen and nutrients between mother and fetus, the establishment of maternal immune tolerance, the defense against pathogens and, in the first stages of pregnancy, the development of the spiral uterine arteries and the proper formation of the placenta [10,11].

The cellular and molecular exchange among mother and embryo in this dynamic microenvironment is immense, and countless interactions take place along it. This way, all the essential functions located in this interface are highly regulated, with any imbalance probably leading to adverse pregnancy outcomes, like RPL [9].

Among the immune cells that populate the maternal–fetal interface, T cells, macrophages, and uterine Natural Killer (uNK) cells stand out. While the first two are more related to the promotion of maternal–fetal tolerance and the establishment of an adequate pro- and anti-inflammatory balance, especially through the participation of certain cell subpopulations (Th1, Treg, Th17…), uNKs are involved in the initiation of placentation and embryo invasion, while also acting as key factors for maternal–fetal compatibility [11,12].

To carry out these processes, crucial for the establishment of a healthy and complication-free pregnancy, uNKs interact with trophoblast cells through their Killer Immunoglobulin-like Receptors (KIR), which find their ligands in Human Leukocyte Antigen-C (HLA-C), expressed by the extravillous trophoblast (EVT) [13,14]. Both molecules show high levels of polymorphism, implying the existence of numerous KIR–HLA-C combinations between mother and embryo [14]. Interestingly, some of these combinations appear to increase the likelihood of miscarriage and adverse reproductive outcomes (pre-eclampsia, fetal growth restriction, preterm birth…), while others are thought to play protective roles against these same events [8,15,16,17,18].

However, the immunological implications of HLA molecules expressed at the maternal–fetal interface go beyond such KIR–HLA-C interactions. In fact, many authors have pointed out possible relationships between RPL and almost all other antigens of the same HLA system, including HLA-G, HLA-DR or HLA-DQ. These observations suggest a relevant involvement of the Major Histocompatibility Complex (MHC) in the achievement of pregnancy.

In this review, we will discuss the functions performed by uNK cells in the maternal–fetal interface, taking into account the effects of the different polymorphisms in the KIR and HLA-C molecules in the uNK-EVT interaction, and their relation to RPL. In addition, we will address the latest evidence on the involvement of other molecules belonging to the HLA system in RPL, and conclude with a discussion of the different therapeutic approaches that are currently recommended in the management of patients diagnosed with this reproductive pathology, and in whom KIR–HLA incompatibility has been discovered.

## 2. The Role of Natural Killer Cells in Early Pregnancy

NK cells represent the most abundant lymphoid population in the uterus (70–90% of lymphocytes), suggesting an important role of these cells in the reproductive sphere. Furthermore, their numbers increase in the endometrium from the follicular to the secretory phase, eventually becoming the main decidual immune cell population during the first trimester of pregnancy [11,12,19].

The functions performed by uNK cells during decidualization and early gestation are related to the support of angiogenesis, the control of trophoblast invasion, and the development of an immune microenvironment conducive to a healthy pregnancy. In fact, one might be surprised that these functions have little to do with the activities that these cells have in the peripheral blood, such as promoting cytotoxic responses against pathogens and cancers [12,20].

In this regard, a differentiation should be made, stating that, in reality, uNKs and peripheral blood NK cells (pbNK) make up two completely different immune populations. This fact, understood today by most physicians, has brought numerous controversies over the years. Caution should be exercised with the measurement and study of pbNKs (which, to this day, can be observed in some fertility clinics) as a tool to draw conclusions about the patient’s reproductive immune status. The lack of consensus regarding the method, threshold, or significance of the results questions the validity of these specific tests as advisable analysis for ART patients [12,21,22].

In this sense, apart from their location, the differences between uNKs and pbNKs are abundant: the former show a high expression of CD56 in their surface, being negative for CD16 (CD56^bright^CD16^−^), whereas the latter express low CD56 and show CD16 molecules (CD56^dim^CD16^+^) [23]. Moreover, pbNKs promote characteristic cytotoxic responses, hardly observed in their uterine counterparts, more inclined to cytokine production [20,24]. Finally, the fluctuation of uNKs throughout the different phases of the menstrual cycle, according to hormonal levels, does not occur for pbNKs, which only increase or decrease their number in response to infections, neoplasms, or other immunological conditions [19,24].

All of this is consistent with the idea that uNK cells are a unique population, very different from any other immune cell, which has been shown to be highly involved in reproduction. In fact, the establishment of a healthy pregnancy occurs early in gestation, at which point the role of uNKs becomes crucial [12,24].

**Figure 1:**
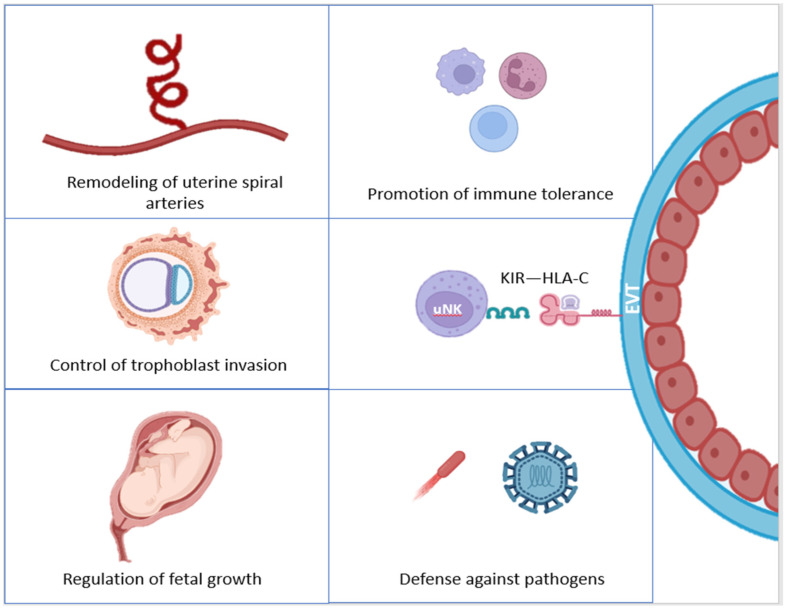
uNK cells’ functions in pregnancy. EVT: extravillous trophoblast; HLA-C: Human Leukocyte Antigen-C; KIR: Killer Immunoglobulin-like Receptor; uNK: uterine Natural Killer cell. Original; created with Biorender.com.

One of the main events in which uNK cells are involved during early pregnancy is the remodeling of uterine spiral arteries. These are branches of the uterine artery that will allow the adequate supply of nutrients and oxygen to the embryo. At the onset of placentation, uNKs promote a series of processes to transform these specific arteries, including: vessel dilatation, increased permeability, the progressive loss of endothelial cells, or phenotypic switching and migration of vascular smooth muscle cells [25,26,27] (Figure 1).

Along with this angiogenic role, uNKs are also involved in controlling trophoblast invasion, preventing exaggerated or poor invasive activity and placentation. This way, their functions contribute to prevent undesirable outcomes, such as ectopic pregnancy. Accordingly, a recent research culturing EVT organoids and primary uNK cells states that this immune cell population is able to promote EVT differentiation by regulating gene pathways involved in epithelial–mesenchymal transition, cell invasion, and cell fusion [12,28] (Figure 1).

These activities, through which uNKs exert fine control over placentation and EVT invasion, are carried out by secretion of cytokines. Indeed, it has been demonstrated that uNKs secrete, in the vicinity of spiral arteries, several pro-angiogenic cytokines, such as Vascular Endothelial Growth Factor-C (VEGF-C), Angiogenin-1 (Ag1), or Angiotensin-2 (Ang2) [29]. The relevance of these angiogenic factors is demonstrated by studies linking certain polymorphisms in the genes encoding some cytokine-associated factors to idiopathic RPL. Therefore, the secretory functions of these cells at the maternal–fetal interface is certainly necessary for this process of vascular remodeling, achieving the correct blood flow for a healthy pregnancy [30].

Likewise, the demonstrated production of Tumor Necrosis Factor-α (TNF-α), Transforming Growth Factor-β1 (TGF-β1) and Interferon-γ (IFN-γ) by uNK cells has been linked to EVT invasion, inhibiting this process, while other uNK products, such as MMP-9 and IL-8, are thought to promote it. Thus, this immune population is able to exert a balanced control over placentation and trophoblastic invasion [26].

In this sense, episodes of ectopic pregnancy or adhesion of the embryo to scar tissue from a previous caesarean section can be partially explained by the absence of decidua and, consequently, of the cells that populate it (such as uNK cells). In these cases, overgrowth and uncontrolled invasion may occur, as the cellular and molecular elements controlling this process are lost [12].

Interestingly, some authors have even observed a pattern of cytokine production, depending on gestational age. In this regard, angiogenic growth factors predominate during weeks 8–10 and, subsequently, from week 12 onwards, the trend changes, and cytokine production to control TVE invasion is favored. This unique switch is further evidence that the role of uNKs during early gestation is essential and finely controlled [26,29].

In this sense, angiogenic processes are promoted by these cells during the first weeks of gestation and, subsequently, this same immune population is responsible for favoring a controlled invasion of the EVT, thus ensuring the establishment of a healthy pregnancy from the beginning [12,26,29]. The occurrence of these phenomena outside the established time frame, as well as exaggerated or insufficient angiogenesis and/or invasion, may compromise the proper development of the pregnancy, possibly leading to undesirable outcomes, such as RPL [12,26].

Beyond these main functions, uNKs also play a role in fetal development, the induction of immune tolerance, and the defense against pathogens. In this sense, through maternal–fetal interactions, uNKs are activated to secrete specific cytokines related to embryonic development, such us pleiotrophin (PTN), osteoglycin (OGN), and osteopontin (OPN). Likewise, uNKs are able to modulate the decidual immune microenvironment, changing the expression of certain receptors, in order to promote immune tolerance towards the semi-allogeneic embryo [12,31].

This specific function concerning immunomodulation of the maternal–fetal interface is of great relevance for a healthy pregnancy. Along with uNKs, other cell populations are also involved in this event, most notably Treg. Indeed, regulatory T cells have been shown to be essential in the promotion of maternal tolerance through the synthesis of several cytokines and thanks, among others, to the activity of FOXP3, a master transcription factor necessary for these cells to fulfil their functions [32,33].

Indeed, dysregulations in this transcription factor or in cytokines produced by Treg have been associated with undesirable reproductive outcomes, such as RPL [34]. Moreover, even specific polymorphisms in both FOXP3 and related cytokines have been linked to an increased risk of RPL, demonstrating the importance of this cell type and the need for balanced immune components at the maternal–fetal interface [35].

Continuing with the functions of uNK cells, in addition to this immunomodulatory activity, shared with Tregs, they are also involved in the defense against pathogens in the uterine environment, being able to recover their cytotoxic capacity in case of infections. This last scenario, together with the promotion of other immune responses by uNKs, can be observed in cases of placental infection by human cytomegalovirus (HCMV), *Listeria monocytogenes*, or ZIKV [31,36,37,38] (Figure 1).

With this, the functions performed by uNKs, which will lay the foundations for a healthy gestation, would be presented. Nowadays it is well known that these functions are regulated, at the maternal–fetal interface, through the EVT–uNK interaction, a binding that is able to activate or inhibit the activities of these important cells [12,24,31]. For this reason, next in this review, we will analyze the main interactions involved in the regulation of uNKs, their effect on the processes in which this cell population is involved and, therefore, the associated reproductive consequences.

## 3. KIR–HLA-C Compatibility and RPL

At the maternal–fetal interface, a bilateral communication is created between the mother and the embryo, decisive for the success of pregnancy. In this milieu, countless cells and molecules are exchanged, and numerous interactions are established. Among them, the most important ones involve Human Leukocyte Antigen (HLA) [14,39,40].

HLA molecules are glycoproteins located on most cell surfaces of the organism, which present a high level of polymorphisms, being, therefore, different from one individual to another. This characteristic explains their involvement in immune responses and, remarkably, in reproduction [41,42]. Different classes and subtypes of HLA molecules have been described throughout the years, and, at the EVT, the following class I HLA antigens can be found: HLA-C, and “non-classic”, HLA-E, HLA-F and HLA-G [43,44,45].

It is remarkable that interactions with uNK receptors have been described for all these molecules. In this sense, HLA-E binds to CD94/NKG2, and HLA-G interacts with members of the leukocyte immunoglobulin-like receptor (LILR) family, the inhibitory or activating receptors ILT2 or ILT4 and KIR, with the latter two also recognized by HLA-F [12,43,46]. However, of all the interactions described in this regard, it is believed thar the one involving HLA-C and KIR is the most relevant in reproductive success, and in particular, in the pathogenesis of RPL [8,12].

KIRs are transmembrane glycoproteins encoded by a cluster of genes located in chromosome 19 [47]. These genes are highly polymorphic, with more than 2000 alleles having been described to date [48,49,50]. Certain possible classifications have been proposed for KIR genes, according to the number of immunoglobulin-like extracellular domains (KIR2D/KIR3D) and the fact that they have a long or short transmembrane tail (KIR2DL/KIR3DL/KIR2DS/KIR3DS) [48,51]. However, the most interesting classification, from a functional point of view, is the one based on the effect that KIRs promote on uNKs, being able to activate or inhibit the activities of these cells [49].

Specifically, KIRs with a short cytoplasmic tail are considered activators, whereas those presenting a long cytoplasmic tail are known as inhibitors [52]. This differentiation has led to the establishment of two haplotypes, depending of the high or low presence of activating KIR genes: KIR B, with mainly activating KIRs, and KIR A, with a predominance of inhibitory KIRs [53].

Added to all this is the fact that HLA-C, the ligand of KIR in EVT, is, again, highly polymorphic, with around 8000 alleles described [54]. However, similarly to the case of KIRs, HLA-C alleles are divided in two categories, according to a dimorphism at the protein level, being classified as HLA-C1, if an asparagine is present in position 80 of the α1 domain, or HLA-C2, if such position is occupied by a lysine [55].

The different possible combinations that can consequently be observed for the KIR–HLA-C interaction at the maternal–fetal interface have aroused the interest of researchers, who wonder whether this polymorphic connection could somehow affect reproductive outcomes. In fact, numerous studies have emerged on this topic, demonstrating a real involvement of KIR and HLA-C in fertility and, specifically, in RPL [8,12,16,17].

When analyzing KIR–HLA-C combinations, it is important to establish that HLA-C1 allotypes bind to inhibitory KIR2DL2 (haplotype B) and KIR2DL3 (haplotype A). For its part, HLA-C2 allotypes can interact with the activating KIR2DS1 (haplotype B) and the inhibitory KIR2DL1 (haplotype A). Interestingly, binding between HLA-C2 and inhibitory KIRs promotes a greater effect than that involving HLA-C1. Thus, HLA-C2 can acts favoring a stronger inhibition of uNKs [56].

Our and other research groups have reported that women of KIR AA genotype (carriers of the inhibitory KIR haplotype only) are the most likely to suffer from RPL and obstetric complications, like preeclampsia or fetal growth restriction (FGR) [17,18,57]. In particular, Hiby and colleagues found a significant increase in the KIR AA genotype frequency in women with history of RPL and preeclampsia [18]. Our group obtained similar results when studying the clinical outcome after single and double embryo transfer (DET) in KIR AA women [57]. Possible explanations for these observations point to impaired functionality of the uNK cell population, thus preventing proper placentation with this phenomenon being reinforced when more than one embryo is present, further complicating maternal immune modulation [17,18,40,57].

Furthermore, and in accordance with the effect of stronger inhibition linked to HLA-C2 allotype, these undesirable reproductive outcomes are enhanced in those KIR AA patients whose embryos carry HLA-C2 alleles, especially if those alleles are ‘foreign’ (paternal or from donated oocytes) [8,15,16,53]. In this regard, some authors reported an increased risk of pre-eclampsia and RPL in KIR AA women carrying fetuses that express HLA-C belonging to the HLA-C2 group [17,18]. Likewise, the specific activating KIR allele for HLA-C2, KIR2DS1, was underrepresented in the population of RPL women [17]. This could serve as further evidence that immune-related RPL could be due to defective placentation, following failed activation of uNK cells [16,17,58].

The last study conducted by our group in this regard showed concordant results, with a higher miscarriage rate and a lower live birth rate (LBR) in a population of KIR AA women facing DETs, compared to KIR AB and KIR BB women. Interestingly, we observed a more pronounced reduction in live birth rate as the fetal HLA-C2 burden increased in these patients [8].

**Figure 2:**
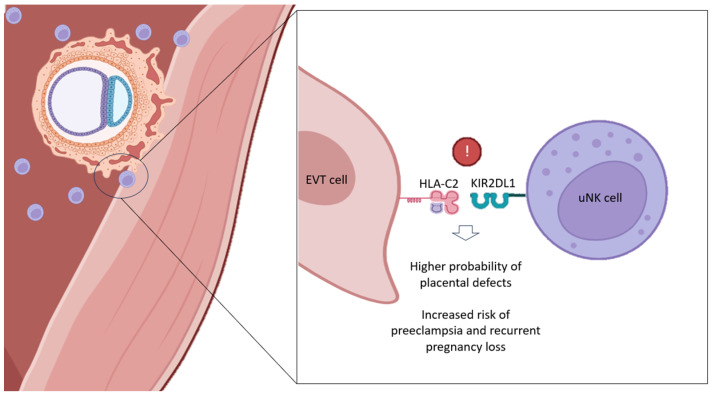
Deletereous effect of specific KIR–HLA-C combinations at the maternal–fetal interface. EVT: extravillous trophoblast; HLA-C: Human Leukocyte Antigen; KIR: Killer Immunoglobulin-like Receptor; uNK: uterine Natural Killer. Original; created with Biorender.com.

According to some authors, the main KIR allele responsible for this immunological incompatibility (KIR AA–HLA-C2) would be KIR2DL1 (located in haplotype A), promoting an excessive inhibitory effect on uNKs when binding to embryonic HLA-C2 at the maternal–fetal interface [53] (Figure 2). On the contrary, the activating KIR2DS1 (located in haplotype B) allele, is supposed to bring certain protection for KIR AA patients and their worst reproductive outcomes. Hence, the significant absence of KIR2DS1 found in women with history of RPL [17]. Supposedly, the KIR2DS1–HLA-C2 combination would be increasing the production of cytokines by uNKs, favoring EVT invasion, uterine remodeling and, consequently, the onset of a healthy pregnancy [58].

Beyond the involvement of KIR AA–HLA-C2 interaction in RPL, other combinations have also been studied. Interestingly, some authors have noted that the presence of certain activating KIRs, such as KIR2DS2, together with a fetus carrying HLA-C1 alleles, can also be associated with a higher risk of RPL [31,47]. Likewise, a decrease in expression of KIR2DL/S1,3 and 5 has been linked to situations of elevated uterine artery resistance index, as a sign of possible placental defects [59,60].

These observations suggest that there must be a tightly controlled balance at the maternal–fetal interface to ensure a healthy pregnancy, and that any dysregulation in EVT–uNK communication towards overactivation or overinhibition could have dire consequences [12,31]. This problem, related to immunological incompatibilities in the couple, is a relatively new scenario for physicians, who sometimes have difficulties when dealing with patients suffering from this issue in the context of ART [24,53].

## 4. Other HLA Molecules at the Maternal–Fetal Interface, and Their Relation to RPL

The maternal–fetal interface is a complex and unique structure, with innumerable interactions and exchanged molecules, all of which are coordinated for the correct maintenance of pregnancy [12,61]. Therefore, it is not surprising that, beyond embryonic HLA-C and its interaction with KIRs, many authors have pointed to other molecules, also belonging to the HLA system, as possible aspects equally associated with RPL. This could further complicate the work of both clinicians and basic researchers in treating RPL, while demonstrating the need for continued research in reproductive immunology [62,63,64].

To begin with, multiple studies highlight the importance of embryonic HLA-G in the proper achievement of placentation and pregnancy. This non-classical HLA class I molecule is expressed in the EVT, and constitutes an important factor in the immunomodulation of the uterine microenvironment, promoting a tolerogenic state conducive to pregnancy. HLA-G has also been shown to play a role in the remodeling of the spiral uterine arteries, thus representing another placentation-related immune factor [65,66]. All these functions are accomplished through the interactions that HLA-G establishes at the maternal–fetal interface with other immune components, including, again, uNK cells [67].

The roles played by HLA-G during early pregnancy are consistent with the results of some authors, who focused on the measurement of soluble HLA-G (sHLA-G). Interestingly, they observed a higher concentration of sHLA-G in pregnant women than in the control group of non-pregnant women. In addition, those who experienced miscarriages during the study had lower (or even undetectable) sHLA-G concentrations compared to both pregnant and non-pregnant women [68]. Although there is some controversy in this regard, with authors reporting different results for sHLA-G levels between pregnant, non-pregnant and RPL patients, most research on this molecule suggests a significant association with healthy pregnancy [69,70,71]. Nevertheless, authors must be cautious when measuring and interpreting results based on sHLA-G concentrations, since this antigen expression could vary according to certain pathologies, like Systemic Lupus Erythematosus (SLE) or Antiphospholipid Syndrome (APS), and even in response to certain medications [71,72,73].

In addition to research on the concentration of sHLA-G, some authors have focused on the study of certain genetic polymorphisms in the non-coding regions of this gene, looking for possible associations with RPL. One of these genetic variants is a 14 bp insertion/deletion located in the 3′ untranslated region (3′UTR) of HLA-G. For years there has been some controversy regarding the relationship of this polymorphism with RPL, with studies reporting a significantly increased risk of recurrent unexplained miscarriages in couples carrying the 14 bp insertion allele of HLA-G [74,75], while some others failed to find such associations [76,77].

More recent publications in this regard include a meta-analysis that does find a higher prevalence of the homozygous genotype for the 14 bp insertion in women suffering RPL [78], and another study that, while not finding a significant association between this polymorphism and RPL, did observe a lower concentration of HLA-G in a group of patients with such an undesired reproductive outcome [79].

Similarly, other research on the possible relationship between genetic variants at the HLA-G locus and RPL seems to find significant associations. In this regard, certain single nucleotide polymorphisms (SNPs) located, this time, in the 5′ upstream regulatory region (5′URR) of HLA-G appear to have a different distribution between women with RPL and control groups. In particular, carriers of SNPs −1179G > A, −725C > G/T and −486A > C showed an increased risk of RPL, with the first two SNPs also being significant in relation to RPL when male partners were also studied [80].

Following the evidence on this gene and reproductive outcomes, it has been reported that the presence of the 3′ UTR haplotype UTR-4 is significantly lower in women with a history of RPL, suggesting a possible role for this haplotype in achieving a healthy pregnancy [62]. Thus, despite initial controversy, much evidence points to a link between HLA-G polymorphisms and RPL. Indeed, many of the genetic variants discussed in this section appear to be capable of altering HLA-G expression and, thus, possibly impairing HLA-G-associated functions during pregnancy, related to immunoregulation and placentation [81,82].

The simplest way to understand the inconsistency between the studies lies in the low polymorphism of HLA-G in the world population: only 158 alleles are described, compared to 8,084 in the case of HLA-C [54]. Much is still unknown about the implications of HLA-G in pregnancy and RPL. There is a need to finally report on polymorphisms that may have a real impact on reproductive outcomes, as well as to gather sufficient evidence on using HLA-G expression in unexplained cases of RPL as a potential therapeutic target to improve outcomes, before starting to find any possible translational solutions to clinical practice [71].

Another HLA molecule also present at the maternal–fetal interface that has been consistently associated with poorer reproductive outcomes and, particularly with RPL, is HLA-DQ. Specifically, a higher proportion of women carrying the HLA-DQ2/DQ8 polymorphism has been observed among RPL patients [83]. Interestingly, this same polymorphism has been associated with an increased risk of celiac disease [84,85] and, in addition, with an increased presence of several autoimmune markers, such as anticardiolipin antibodies and anti-thyroid peroxidase antibodies [86].

The already-known association between autoimmune diseases and RPL [87,88,89,90,91] serves as an explanation for the link between this HLA-DQ polymorphism and spontaneous abortions, as a reminder of the importance of an adequate immunological environment for a complication-free pregnancy.

Research on the involvement of HLA in RPL continues, with all types of HLA molecules being studied in this respect. Thus, numerous other HLA alleles have shown a significant association with RPL, such as the HLA-DRB1*07 allele [64] or the HLA-B13 allele, as well as the “shared HLA” phenomenon, referring to alleles shared by both partners, which, in turn, seem to increase the risk of RPL [92].

However, the involvement of HLA class II molecules (beyond the association with autoimmune pathologies described above) is more difficult to explain, as EVT cells do not express any of them. EVT expresses the class I HLA molecules described, but not class II HLA molecules [43,45,93]. Therefore, the use of HLA-DQ/DR typing in the couple to define a degree of “incompatibility” has little scientific support at present.

Although maternal–fetal compatibility has usually been studied in the context of KIR–HLA-C interactions, all the research discussed in this section demonstrates the involvement of many other HLA molecules, much less studied in this regard, in the achievement of pregnancy. Continued research on this topic will shed light on the complex involvement of the maternal immune system in reproduction and, as in the case of KIR–HLA-C, begin to find possible approaches to reduce immune-related RPL.

## 5. Management of Patients with Immunological Incompatibility

Until very recently, immunotherapies for patients suffering from recurrent miscarriages were based on a supposed cytotoxic/proinflammatory activity of uNKs. In fact, the role of these cells during pregnancy was, in many cases, misunderstood, with some authors proposing them as a direct cause of RPL, through the promotion of inflammation in the uterine microenvironment and trophoblast cell damage [94,95].

This view of uNKs, so far from their role as drivers of angiogenesis and placentation, led to the introduction of immunomodulatory therapies in patients to reduce the action of these cells. Moreover, immunomodulators were sometimes used with the sole criteria of “NK cell increase”, without developing further immunological tests in patients or taking into account their KIR–HLA compatibility [12,24,40].

Most of these immune therapies, being of questionable usefulness, focused on minimizing possible proinflammatory states in patients, while reducing NK cells activity. They included steroids, heparin, aspirin, intravenous injection of immunoglobulin G (IVIG), paternal leukocyte immunization, TNF-α inhibitors, intralipids, granulocyte colony stimulating factor (G-CSF) and progesterone (Table 1) [12,22].

To begin with, the use of glucocorticoids in women undergoing ART has yielded controversial results [96]. In this sense, two meta-analyses reported positive outcomes when administering this type of medication for RPL patients [97,98], although the authors cautioned about the heterogeneity of the included research. In contrast, some researchers found no improvement in reproductive outcomes following the use of corticosteroids, such as prednisolone [99,100] (Table 1). However, it should be noted that one of these studies did not perform patient selection based on a history of RPL, and used prednisolone in combination with other putative therapies, such as aspirin and doxycycline [100].

It is also noteworthy that, in this study, a reduction in LBR was observed after frozen embryo transfer in the treated group [100]. This may serve as a warning to physicians to be cautious about introducing specific medication into IVF cycles. In addition, the use of corticosteroids has been associated with several side effects such as prematurity, orofacial clefts, gestational diabetes, and hypertension [101,102].

Finally, a more recent meta-analysis concluded that there was insufficient evidence that glucocorticoid administration in assisted reproduction patients actually influenced reproductive outcomes [103] (Table 1). However, research with regard to the introduction of glucocorticoids in IVF patients is characterized by the heterogeneity of investigations and the lack of standardization in terms of the patient groups, form of administration, time of use, or dosage employed, which currently makes it difficult to compare between studies and to identify specific groups of women who could benefit from this medication [22,96].

On the other hand, treatment with heparin and/or low-dose aspirin has not been supported by the most recent RCTs and meta-analyses, with the exception of antiphospholipid syndrome [6,104,105,106,107,108,109] (Table 1). These studies showed no significant improvement in reproductive outcomes in patients with RPL and/or pregnancy complications (except for the benefit of aspirin in lowering the risk of pre-eclampsia and FGR) [104,105,106,110].

Likewise, studies regarding the use of IVIG in women suffering RPL have found no improvement in the live birth rate (LBR), even pointing out to some potential adverse events after the use of this therapy [106,111] (Table 1). Allogeneic lymphocyte immunotherapy (LIT) involving “paternal immunization” is also inadvisable, given the inherent risks of potentially serious complications that the administration of blood products always entails [6]. Although certain authors observed some initial potential benefits when patients with RPL were treated with LIT [112] (Table 1), this putative therapy was eventually banned by the U.S. Food and Drug Administration (FDA) due to several safety concerns [22], with some authors claiming to have found no actual improvement in pregnancy outcome after use [113].

With respect to TNF-α blockers, some studies seem to observe possible advantages of using Adalimumab (anti-TNF-α), along with other therapies, such as heparin, LDA and/or IVIG in ART patients, revealing improved clinical outcomes (implantation, clinical pregnancy, live birth) in the treated group [114,115] (Table 1). Nevertheless, caution should be exercised in this regard, as no RCTs have been found to support the use of Adalimumab (monotherapy or combined) in healthy ART patients, with the current evidence based on a few observational studies [6,116]. Moreover, some research has even emerged warning about the possible general risks [117] and poorer reproductive outcomes in patients with rheumatoid arthritis under the use of this therapy [118] (Table 1).

The situation is similar with the use of intralipids as a possible therapy against RPL. While some studies seem to record some benefit in clinical outcomes (although not statistically significant) [119], others do not find any improvement, and even point to the possible harmful effect of this supposed treatment [120] (Table 1). It should be noted that this last prospective cohort study had to be prematurely terminated after failing to achieve a single birth in the intralipid-treated group, whereas in the placebo group the LBR was better than in the intralipid group, being close to 30% [120]. In addition, the absence in the scientific literature of well-designed randomized studies currently discourages the use of intralipids in ART patients [6,116].

For its part, the introduction of G-CSF for patients with a history of RPL could be supported according to a RCT in which LBR increased drastically in the group treated with this cytokine [121] (Table 1). However, again, some controversy arose regarding the usefulness of this immunotherapy with the publication of an RCT that showed no evidence of improved clinical outcomes after G-CSF injection in healthy patients with no immune factor [122] (Table 1). In this scenario, to successfully address the usefulness and safety of this immunomodulatory therapy, it would be advisable to consider RCTs with a large number of patients and taking into account the potential immunological factor, in order to better understand the possible mechanism of action of G-CSF [122].

Interestingly, that is the case for some research, including cohort studies and RCTs, who had examined the clinical consequences of G-CSF injections in RPL patients, recruiting a higher number of subjects [123] and analyzing KIR genotypes [124]. Indeed, both showed better clinical outcomes, in terms of LBR and pregnancy rate, when administering G-CSF, pointing this molecule as a promising therapy for RPL patients with inhibitory maternal KIR [123,124] (Table 1).

Furthermore, a recent study including RPL patients with KIR–HLA-C mismatch supported the safety of this treatment, finding no significant differences when studying possible perinatal complications. Therefore, the implementation of G-CSF appears to be a safe and useful option for patients with RPL with possible altered maternal–fetal communication, due to KIR–HLA-C risk combinations [125].

Finally, many physicians have advocated for the administration of progesterone to prevent possible miscarriages of immune origin, due to the role of this hormone in inducing secretory endometrial changes and possibly promoting a favorable immunological milieu [6]. Although one of the most important projects in this regard, the Progesterone in Recurrent Miscarriage (PROMISE) trial, showed no difference in clinical outcomes when comparing the treated group versus placebo [126], a subsequent meta-analysis of several RCTs did observed better results in terms of miscarriage and live birth rate after progestogen administration [127] (Table 1).

Later, a network meta-analysis involving 5682 women was carried out, with the aim of asses the relative effectiveness and safety profiles for different progestogen treatments used in patients with history of RPL. Based on the conclusions reached in this meta-analysis, the available evidence suggests that progestogens are likely to make little or no difference in the LBR in these patients. However, with respect to vaginal micronized progesterone administration, a possible increase in the LBR could be observed in women with a history of one or more previous miscarriages and bleeding in early pregnancy [128] (Table 1).

**Table 1:** Main research regarding the immunomodulatory therapies discussed, including patient selection and results/conclusions of each individual study.

Immune Therapy	Patients	Results/Conclusions	Publication
Glucocorticoids	Women with history of idiopathic RPL	Prednisolone therapy improves pregnancy outcomes in women with idiopathic RPL	[97]
Glucocorticoids	RPL and implantation failure patients	Significant positive effect of prednisolone was found	[98]
Glucocorticoids	Women with RPL and RIF	No evidence for a significant beneficial effect for prednisolone therapy on pregnancy outcomes.	[99]
Glucocorticoids, combined with aspirin and doxycycline	Women undergoing IVF/ICSI with own oocytes	No benefit of this combined adjuvant strategy in fresh IVF cycles, and possible harm when used in frozen cycles	[100]
Glucocorticoids	Subfertile women undergoing IVF or ICSI	There is insufficient evidence that administration of peri-implantation glucocorticoids in IVF/ICSI cycles influenced clinical outcomes	[103]
Heparin and aspirin	Women with unexplained RPL	No significant differences in live birth rate were found	[106]
Heparin and aspirin	Women with unexplained recurrent miscarriage	Neither aspirin combined with nadroparin nor aspirin alone improved the LBR	[104]
Heparin	Patients with history of placenta-mediated pregnancy complications	Low-molecular-weight heparin does not seem to reduce the risk of recurrent placenta-mediated pregnancy complications	[105]
Aspirin	Women at risk of preeclampsia	Low-dose aspirin is an efficient method of reducing the incidence of preeclampsia and FGR	[110]
IVIG	Women with unexplained RPL	Intravenous immunoglobulin showed no effect on live birth rate compared with placebo	[106]
IVIG	Women with history of RPL	No significant difference in the frequency of live birth was found	[111]
LIT	Women with history of RPL	Beneficial effect of the use of immunotherapy with lymphocytes in cases of RPL	[112]
LIT	Women who had had three or more spontaneous abortions of unknown cause	Immunization with paternal mononuclear cells does not improve pregnancy outcome in women with unexplained recurrent miscarriage	[113]
LIT	Women with history of RPL	Lymphocyte immunotherapies do not have the required FDA approval and are considered investigational drugs	[22]
TNF-α inhibitor	Women with infertility and T helper 1/T helper 2 cytokine elevation	The use of a TNF-α inhibitor and IVIG significantly improves IVF outcome	[115]
TNF-α inhibitor	Patients with rheumatoid arthritis	The rate of spontaneous abortion was highest among patients exposed to anti-TNF at the time of conception	[118]
Intralipids	Women with unexplained secondary infertility, RPL, and elevated levels of natural killer cells	Intralipid supplementation did not increase frequency of chemical pregnancy.	[119]
Intralipids	Women aged 40–42 years with a previous history of miscarriage	The use of intravenous intralipid to suppress natural killer cell activity does not seem to improve the chance of a live delivery	[120]
G-CSF	Women with RPL	Improved LBR and pregnancy rate in the group treated with G-CSF, compared to control group	[121]
G-CSF	Patients under 40 years old with at least two unexplained pregnancy losses	No significant difference was observed in terms of chemical pregnancy, implantation, clinical pregnancy and abortion	[122]
G-CSF	Women with RPL	Improved LBR in the group treated with G-CSF, compared to control group	[123]
G-CSF	Women with RPL/RIF	There is evidence that G-CSF increases the pregnancy rate in ART treatment, particularly in patients with RIF, and decreases the abortion rate in patients with RPL	[124]
Progesterone	Women with unexplained RPL, aged between 18 and 39 years at randomization, conceiving naturally	There is no evidence that first-trimester progesterone therapy improves outcomes in women with a history of unexplained RPL	[126]
Progesterone and progestins	Women with a history of unexplained recurrent miscarriage	Supplementation with progestogens may reduce the incidence of recurrent miscarriages and seem to be safe for the fetuses	[127]
Progestogens	Women with a history of one or more previous miscarriages and early pregnancy bleeding	Vaginal micronized progesterone seems to increase the live birth rate	[128]

Likewise, these and other authors point out the current limitations for selecting a specific regime of progestogens for patients undergoing ART, since, in the current scientific literature, multiple formulations, dosages and administration routes can be found, in addition to other differences between studies, such as the criteria for patient selection. Further studies to confirm the effectiveness and safety of the different progestogen treatments are needed, before definitively implementing them for threatened and recurrent miscarriage [6,128].

As can be seen, the use of immunotherapy in patients with a history of RPL, of presumed immunologic cause, is fraught with inconsistencies, probably due to high heterogeneity of trials and patients selected. Although some proposed treatments appear to have potential benefits (such as administration of G-CSF or vaginal micronized progesterone), more well-designed studies are needed before implementing any of them in routine clinical practice. Moreover, most of these so-called therapies are not without risk, as many of them are associated with significant side effects [6,12,96].

Furthermore, not only is it important to reach consensus on the efficacy and safety of these therapies, but it is also necessary to identify specific patients who might benefit from any immunotherapy in the context of ART. For example, prophylactic prescription of heparin and low-dose aspirin, although it does not seem to improve clinical outcomes in patients with RPL in general [6], is indeed recommended for women diagnosed with anti-phospholipid (APS) with history of RPL or obstetric complications as preeclampsia, stillbirth, FGR, and preterm birth [109].

This demonstrates the importance of thorough testing of patients before applying any immunotherapy. The sole criteria of an increase in the number of NK cells should not be a reason to apply this type of therapy since, as already explained, it has not been shown to be a direct cause of RPL. Perhaps the increase in this cell population could be giving clues of an altered immune milieu, related to undesirable reproductive outcomes, but it should not be used as the only reason to treat these patients with any of the above immunotherapies [12,129].

uNKs are critical in reproduction, and we should all understand their actual role in pregnancy before attempting to alter their number or functions. In fact, one of the main aspects of uNKs related to RPL, KIR–HLA-C compatibility, is completely underestimated in the search for immunotherapies, as none of the treatments already explained focused on this issue. According to some authors, the best management of patients with this immunological incompatibility could be carried out through two main approaches: donor selection and limiting the number of embryos to be transferred [8,12,24].

**Figure 3:**
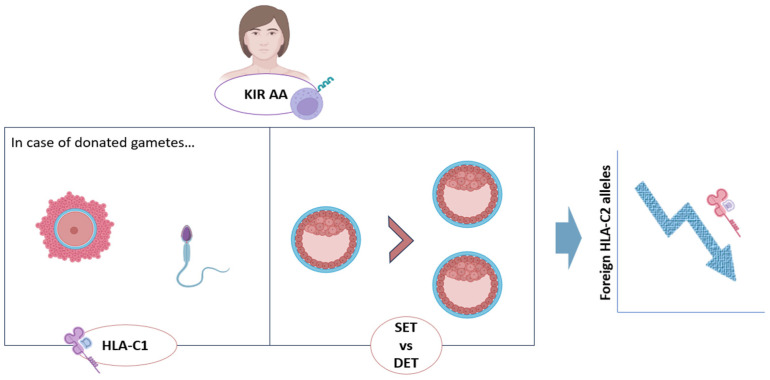
Proposed management of KIR AA patients in the ART setting, in order to decrease the HLA-C2 allelic load to which they could be faced in pregnancy [8]. DET: double embryo transfer; HLA-C: Human Leukocyte Antigen-C; KIR: Killer Immunoglobulin-like Receptor; SET: single embryo transfer. Original; created with Biorender.com.

Both of these approaches are based in the observations that the risk of miscarriage or obstetric complications in KIR AA women increased with the number of HLA-C2 alleles carried by the embryo, especially if they are of ‘foreign origin’ (from an oocyte donor or paternal) [8,53]. In this sense, two studies including patients with history of RPL or RIF showed higher miscarriage rate and lower LBR after double embryo transfers (DETs) in KIR AA mothers compared with the other KIR genotypes. In addition, a significant decrease in LBR was registered as the fetal HLA-C2 load increased in KIR AA women. Therefore, it seems that single embryo transfer (SET) could be the best option for KIR AA patients, reducing this way the potential number of HLA-C2 alleles and improving clinical outcomes for these women [8] (Figure 3).

Furthermore, for those KIR AA patients undergoing IVF with donated oocytes, donor selection according to HLA-C genotype could be advised, preferentially choosing HLA-C1C1 donors for these patients with inhibitory KIR genes (Figure 3). This is an inexpensive and low-risk intervention that, if equally available in assisted reproduction centers, could help to improve clinical outcomes in these patients, possibly decreasing the possibilities of miscarriages [12,24].

Future research on uNK cells, their functions, and the impact that their KIR receptors together with HLA ligands may have on pregnancy, could open the door to new approaches in the prevention of pregnancy disorders. Similarly, the study of RPL patients, not only in terms of uNKs but in any other immunological aspect related to this reproductive condition, could help physicians to find the best therapy for them, always from the understanding of the important role that the immune system plays in reproduction [12,39,53].

## 6. Discussion

For decades, the involvement of immunology in pregnancy has been underestimated and misunderstood by many clinicians and basic researchers. The shared idea that immune cells might act by recognizing the embryo as a foreign identity, activating themselves to attack and kill trophoblast cells has been around for years [12,24]. In particular, these ideas focused on NK cells, assuming that their cytotoxic capabilities recorded in the peripheral blood could also translate to the uterine microenvironment [94].

In this scenario, several tests emerged with the aim of measuring the number of NK cells in patients, assuming that an increase in this cell population could explain previous miscarriages or other reproductive conditions suffered by these patients. In fact, several authors reported observing a high percentage of NK cells with a cytotoxic profile in the endometrium of women with a history of RPL [95,129,130].

Thus, women in whom such a cellular increase was recorded began to be treated with supposed immunomodulatory therapies to reduce the amount of NK cells, as well as their cytotoxic properties and, therefore, to preserve the embryo from maternal immune attacks. These therapies have been discussed throughout this review and, as mentioned above, most of them offered inconsistent results as to their real benefit for ART patients, lacking RCTs evaluating their efficacy, and being linked to significant side effects, with no proven safety either [6,12,96].

Fortunately, in this scenario of uncertainties, some researchers in the field began to question the real role of NKs, along with other immune cells, in pregnancy, pointing out the lack of evidence regarding the alleged immune attacks on embryonic cells, as well as the usefulness of the immune therapies that were administered to patients, who were already in a delicate situation, burdened with having gone through several miscarriages. In fact, it soon became clear that these supposed immunological reactions against the embryo did not take place, and that a deeper understanding of the functions of NK cells during pregnancy was necessary before treating patients from an immunological perspective [12,13,24,53].

Not only did the treatment have to be revised, but also the tests offered to patients. In this sense, these authors wish to highlight that, with regard to the measurement of NK cells, there was no agreed cut-off point to determine which NK cell values should be considered outside normality, nor was there a standardized protocol to be followed for patients in whom immunological imbalances were discovered. Moreover, there was not even a differentiation between pbNKs and uNKs, with both populations usually measured as a whole [12,21,24].

Today, research in reproductive immunology has progressed dramatically and, as a result, we have gained a better understanding of the role of uNKs in reproduction and their implication in maternal–fetal compatibility. In this regard, several experts in the field have come to the conclusion that NK cell assays could be offered to patients, but only as a research tool for women with a history of RPL, which simply provides extra information on an individualized basis, and not as a diagnostic test that can be used to apply specific treatments by itself [12,21,22,131]. In fact, the increase in the percentage of cytotoxic NK cells could be a sign of immune imbalance in these patients, which could provide clues as to the treatment to be selected for these women, but not the direct cause of the miscarriages suffered [129,131].

In this sense, current knowledge about uNK cells has allowed us to understand the essential implication that these and other immune cells have in the establishment of pregnancy, being involved in processes such as the promotion of angiogenesis or the control of trophoblast invasion, thus favoring adequate placentation and a healthy gestation from its onset. This is the reason to be cautious when trying to affect the number or activity of these cells, since we would be affecting crucial events in reproduction [12,21].

These functions of the uNK cells are, moreover, strictly regulated, and take place thanks to the interactions that these immune cells establish with the trophoblast cells. In particular, the interactions between KIR, expressed by uNKs and HLA-C molecules, their ligands in the embryo, have been of interest in this respect. Through this maternal–fetal communication, placentation is regulated and numerous aspects of pregnancy, like fetal growth, protection against pathogens or induction of immune tolerance are favored [12,31].

Interestingly, a high level of polymorphism has been observed in this KIR–HLA-C interaction, leading to a difference in the degree of uNK activation, depending on the alleles involved. This issue aroused the interest of researchers in this field and led to a large number of studies to evaluate possible deleterious KIR–HLA-C interactions, which could be related to undesirable reproductive outcomes, such as RPL. In fact, it was soon observed that, among others, the interaction established in women KIR AA (inhibitory KIRs), when the embryo carried HLA-C2 alleles, was of concern, with these cases being linked to a higher probability of spontaneous abortions, FGR or preeclampsia, probably due to a lack of activation of uNKs, and their being prevented from performing the important functions attributed to them [8,15,17,18].

This discovery helps to change the concept that physicians have hitherto had of uNKs and changes the perspective from which these cells should be studied and understood. The enormous variety of KIR–HLA-C interactions, as well as the effect that each of them could have on reproductive success, should be further investigated. Moreover, measures should be explored to appropriately manage those patients who suffer from immunological incompatibilities of this type [12,21].

To date, proposed approaches include limiting the number of embryos transferred and introducing HLA-C into donor selection criteria, always with a view to reducing the burden of foreign HLA-C2 alleles to which a KIR AA woman might be exposed. This approach to donor selection could be beneficial for RPL patients, and would be easier to introduce in clinical practice than any other alternative way of favoring specific HLA-C alleles, as embryo selection could be [8,12,53,57].

However, the complexity of the maternal–fetal interface and its related immunologic compatibility does not end with HLA-C. In fact, other HLA molecules expressed by the embryo have been similarly linked to undesirable reproductive outcomes, such as RPL. Among these, HLA-G, which also binds to uNKs at the maternal–fetal interface, seems to stand out. According to the latest publication, its concentration and genetic variant are of interest in this regard [71]. Likewise, the relationship between some HLA alleles and certain autoimmune disorders should be further studied as another possible factor involved in miscarriages [86].

The impact of immunology on the pathogenesis and diagnosis of RPL is enormous and varied. Immunological testing of patients should be performed to rule out possible diseases beyond uNKs (such as APS), and KIR–HLA-C testing could also be performed to control, to the extent possible, for any immunological compatibility. In this sense, RCTs are needed to demonstrate the importance of donor selection in KIR AA patients, while continuing to investigate the rest of HLA-related interactions and their implication in reproduction which, so far, seems to be quite considerable [12,31,39,53].

## 7. Conclusions

Reproductive immunology is a complicated and extensive subject, whose research has been going on for decades. In this aspect, it has been demonstrated that immune communication between the mother and the embryo is essential in the control of several key aspects of pregnancy (immune tolerance, angiogenesis, fetal growth…) [31,39].

In particular, the interactions that exist between uNK cells and trophoblast cells through KIR–HLA interactions have been pointed out as one of the main regulators of uNKs activity and, therefore, of these important events. Women with mainly inhibitory genes for these KIR receptors seem to have a worrisome prognosis when facing HLA-C2 embryos, possibly promoting this situation an exaggerated inhibition of uNK cells functions. These women suffer more frequently from reproductive conditions such as RPL, as well as various obstetric complications in pregnancies that could be at risk from their very beginning [8,12,17].

KIR and HLA-C testing in patients with a history of RPL could help provide a potential explanation for these miscarriages and their management with current expert advice in the field (donor selection and SET vs. DET) could improve the clinical outcomes of these patients suffering from RPL, probably of unknown etiology to date. Understanding the pathophysiology of immune-mediated RPL as well as the role of uNKs and other related immune cells and molecules in pregnancy, is mandatory to treat patients appropriately, moving away from previous theories of “immune rejections” and their supposed associated therapies [6,8,12].

## 8. Future Directions

Research on reproductive immunology and its involvement with undesirable conditions such as RPL is gaining importance. However, the study of this topic should be encouraged among clinicians and basic scientists to further clarify the many unknowns that still exist about the involvement of the immune system in pregnancy and, in particular, of uNK cells and HLA molecules. The interactions that these cells establish, not only with the embryo during pregnancy, but even before the arrival of the blastocyst, are innumerable, present a high level of polymorphism and raise numerous questions regarding their implications in pregnancy, questions that must be clarified in the coming years through scientific research [13,39,43].

Likewise, physicians should be educated on how best to manage patients with a history of RPL of presumed immunologic cause. It is recommended to investigate KIR–HLA C status in these patients to understand any possible maternal–fetal incompatibility. Carefully studying each couple and selecting an effective and safe treatment on a case-by-case basis might increase the reproductive success of the patients [12].

Finally, the introduction of HLA-C in the donor selection criteria for KIR AA patients still requires confirmation through appropriate RCTs as, to the best of our knowledge, all data is retrospective and/or observational and, thus, subject to biased patient selection. This donor selection could improve the prognosis of patients with RPL, and would be a very easy approach to introduce in current ART protocols, finally achieving the understanding of the importance of immunology in RPL [8,12].
